# A new dawn for the use of traditional Chinese medicine in cancer therapy

**DOI:** 10.1186/1476-4598-8-21

**Published:** 2009-03-20

**Authors:** Harendra S Parekh, Gang Liu, Ming Q Wei

**Affiliations:** 1 The University of Queensland, School of Pharmacy, St Lucia QLD 4072, Brisbane, Australia; 2 Division of Molecular and Gene Therapies, School of Medical Science – Griffith Health, Gold Coast Campus, Griffith University, Brisbane, QLD 4222, Australia

## Abstract

Although traditional Chinese medicine has benefitted one fifth of the world's population in treating a plethora of diseases, its acceptance as a real therapeutic option by the West is only now emerging. In light of a new wave of recognition being given to traditional Chinese medicine by health professionals and regulatory bodies in the West, an understanding of their molecular basis and highlighting potential future applications of a proven group of traditional Chinese medicine in the treatment of a variety of cancers is crucial – this is where their calling holds much hope and promise in both animal and human trials. Furthermore, the rationale for combining conventional agents and modern biotechnological approaches to the delivery of traditional Chinese medicine is an avenue set to revolutionize the future practice of cancer medicine – and this may well bring on a new dawn of therapeutic strategies where East truly meets West.

## Introduction

Reports of therapeutic success with traditional Chinese medicine (TCM) have until very recently been met with much scepticism and pessimism by the West, due in-part to the sheer lack of available credible and rigorous clinical data and at claims that a given TCM can remedy common ailments and be just as efficacious in eliminating life threatening diseases, such as cancer. The tide is now beginning to turn on this negative outlook, aided by the ever-increasing migration of people and along with them knowledge (based upon ancestral cultural influences) from two of the world's fasting growing populations, China and India, to the West [1]. This translation to the West of ancient complementary and alternative medicine formularies and their ever-increasing integrative role in the armoury against cancer means that their presence and place in modern medicine can no longer be overlooked, by regulatory authorities and clinicians alike, as being merely anecdotal.

**Figure 1:**
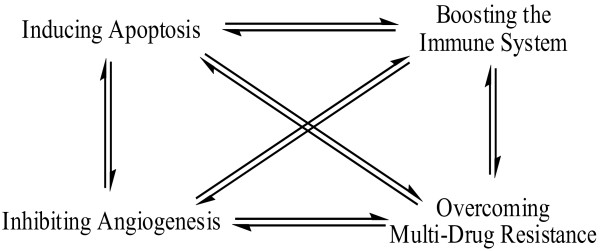
**The complex interplay between the primary mechanisms of TCM**.

The age-old holistic approach employed by Chinese practitioners proposes that a multitude of events are key to returning a patient to a healthy state; where cancer therapy is concerned these primarily include an interplay between the induction of apoptosis/cell-cycle arrest, inhibition of angiogenesis, overcoming multidrug resistance (MDR), and boosting the immune system (Figure 1). Following an extensive review of the literature we describe the detailed molecular basis of a proven group of TCM, and highlight reported synergies when administered alongside so-called 'conventional therapies' in tumour cell regulation and in bringing about homeostasis.

The many physiological growth control mechanisms that regulate cell proliferation and tissue homeostasis are linked to apoptosis and it follows that resistance developed to this '*programmed cell death*' can be directly linked to prolonged tumour cell survival and resistance to therapy [2,3]. The processes resulting in apoptosis are mediated by extrinsic (*via *death receptors) or intrinsic (*via *mitochondrial) pathways and although their paths are not mutually exclusive, evidence suggests the latter certainly predominates where TCM is concerned [4]. Here we look in some detail at two of the primary mechanisms – '*apoptosis*' and '*angiogenesis*' in the context of TCM, proposed to be key avenues responsible for imparting therapeutic efficacy against a wide range of cancers.

### Molecular basis of TCM – Apoptosis

Apoptosis is guided by a range of complex multi-step, multi-pathway programs that eventuate in the breakdown of cellular DNA leading to cell death [5]. And by far the most emphasized and reported endpoint in TCM trials to-date have been those of cell cycle arrest and apoptosis [6,7]. The cascade of intracellular events triggering cell death have been identified to involve activation or suppression of a number of key receptors, genes and enzymes [8]. The mitochondria, a cell's energy source, is recognized as playing a central role in the sustained survival of cells and many of the triggers to apoptosis are known to act here, either directly or indirectly [9,10].

#### Apoptosis by the 'Caspase' effect

Of the pro-apoptotic enzymes implicated in TCM activity the family of cysteine proteases, commonly termed '*caspases' *are key players with their role and function extensively reviewed elsewhere [2,3,10]. Briefly, caspases are divided into two broad groups, the 'initiators' and the 'executioners' and once activated they go on to activate other pro-caspases that trigger apoptosis. The primary role of 'initiator' caspases (caspase-8, -9 & -10) is the processing and activation of both pro-enzymes (procaspase-8, -9 & -10) and 'executioner' caspases (mainly caspase-3, -6, & -7) [2]. Pro-caspases are inactive forms of their cousins, the caspases, and any processing of these pro-enzymes is regarded as a reliable marker for caspase activation, and so apoptosis. It is the result of 'executioner' caspases cleaving each other that triggers an amplifying proteolytic cascade, effecting cleavage/degradation of cellular substrates – these aptly named 'death' substrates are responsible for signalling biochemical and morphological changes that eventuate in cell death [11].

#### Apoptosis by NF-Kb

Cellular stress is invoked by an imbalance in a cell's redox state. Nuclear factor-κB (NF-κB) is a key regulatory molecule, activated when a cell experiences oxidative stress [12]. It is well-documented that tumour necrosis factor-α (TNF-α) and the closely related tumour necrosis factor-related apoptosis ligand (TRAIL) along with lipopolysaccharides, interleukins (IL) and UV or IR radiation all impart cellular stress that triggers the NF-κB cascade [13,14]. NF-κB ordinarily resides in the cytosol of non-stressed cells, non-covalently bound to the family of inhibitory-κB (IκB) proteins which function to mask the nuclear localization signal (NLS) present on NF-κB. When the redox balance of the cell is perturbed by extracellular stimuli, IκBs are rapidly degraded exposing the NLS. The consequence is transfer of NF-κB to the nucleus where it regulates gene expression, the products of which are directly involved in tumorigenesis [15,16].

#### Apoptosis by TCM

Numerous studies have been aimed at deciphering the precise molecular basis for a variety of TCM and those most noteworthy are discussed below.

**Figure 2:**
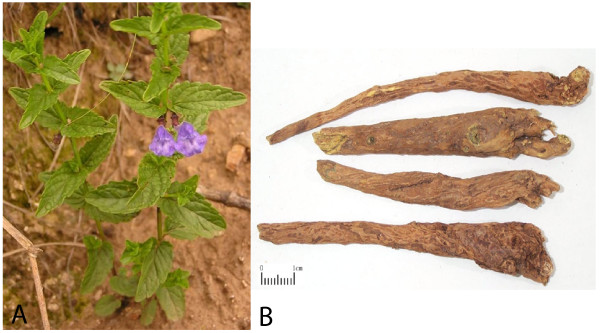
**Plant (A) and root (B) of *Scutellaria Baicalensis *('golden root') where root scale is shown as 1 cm**.

The root of *Scutellaria Baicalensis *(Figure 2), commonly referred to as '*Baikal skullcap*', '*Huang qin*' or the '*Golden Root*', is probably one of the most widely used herbs in TCM preparations with its flavonoid-rich elements considered to impart anti-inflammatory, anti-viral, anti-bacterial and anti-neoplastic activity [17-20].

It is also one of the most widely studied by researchers, being used either alone or more often in combination with other TCM for a range of cancers (*in vitro *and *in vivo*), namely of the prostate, breast, lung, liver and ovaries [6,21-23]. The roots chief therapeutic ingredient is *baicalin*, although it is converted to *baicalein *by intestinal gut flora with this latter bioactive firmly considered to be the primary active constituent responsible for its pro-apoptotic and anti-proliferative effects [21]. Extensive studies reveal that other flavonoids present within the herb e.g. neobaicalein, wogonin and wogonoside are also at work and that their co-synergies most likely contribute to the observed efficacy [24]. The principle mechanism of action of *Scutellaria Baicalensi *is *via *the inhibition of eicosanoid synthesis – being important mediators of pro-inflammatory (cyclooxygenase-2 (Cox-2)) and tumour cell proliferatory (lipoxygenase) markers [25]. Simultaneous inhibition of Cox-2 and 12-lipooxygenase has been shown to result in both reduced inflammation and tumorigenesis [25,26]. The role of *baicalein *against cell proliferation in PC-3 and DU145 prostate cancer cell lines is that of cell cycle arrest (at G_0_-G_1_) while also inducing apoptosis – confirmed *via *detection of caspase-3, at concentrations typical for administration to humans [27].

Further evidence of TCM acting *via *caspase activation and NF-κB also exists with Takrisodokyeum (TRSDY) – comprising 12 herbs in various proportions [28]. Caspase-3 activity assays conducted by Kwon *et al *on promyelocytic leukeamic cells (HL-60) cells pre-treated with TRSDY revealed that apoptotic cell death was indeed caspase-3 induced [27]. Its activation resulting in classical apoptotic signs including DNA fragmentation, chromatin condensation and plasma membrane blebbing [29,30]. Introduction of a caspase-3 inhibitor resulted in no detectable caspase-3 production and the downfield cleavage of cellular death substrates was also absent. It was further identified that oxidative stress by way of hydrogen peroxide generation was a co-contributor to apoptosis in the same population of cells. In an attempt to elucidate which of caspase-3 or hydrogen peroxide was generated first, cells were pre-treated with an antioxidant and scavenger, quenching any oxidative agent. Levels of caspase-3 were found to be negligible in this case, strengthening the hypothesis that caspase-3 activation does not take place in these cells without prior oxidative stress [31,32]. Whether intracellular events leading to apoptosis follow this sequence of events in other cancer cell lines using this or other TCM is yet to be determined although parallel studies using other TCM do concur that activity is *via *selective members of the caspase family, namely caspase-3 [33-36].

TRAIL has received considerable attention by researchers since the discovery that most cancer cells are sensitive to its apoptotic effect but that normal cells confer resistance to it [37]. The potential of TRAIL as a realistic future therapy against cancer was further encouraged by the discovery that conventional therapy, namely chemotherapy or γ-irradiation can sensitize cells previously resistant to TRAIL [38-40]. The bioactive Triptolide (PG490) – extracted from the TCM *Tripterygium wilfordii *has been studied extensively for anti-inflammatory and immunosuppressive activity, being shown to sensitize various types of tumour cells to apoptosis induced by TRAIL, TNF-α and chemotherapy [41-44]. A study conducted by Frese *et al *evaluating PG490 suggests that it sensitizes previously resistant Calu-1 lung cancer cell lines to TRAIL-induced apoptosis while sparing normal human bronchial epithelial cells [42]. PG490-mediated sensitization of the cells to TRAIL requires activation of a family of extracellular-regulated protein kinases (ERK's), namely ERK-1. Located in the intracellular environment ERK-1 is thought to be the crucial link bridging the process that follows death receptor activation and precedes caspase activation. TRAIL, acting *via *death receptors (TRAIL-R1 & R2) forms a death-inducing signalling complex (DISC); this then recruits an 'initiator', caspase-8, which begins a cascade of protease activation enlisting the 'executioner' – caspase-3, promoting cleavage of death substrates with the end-point being cell death. [45-47]. Carter *et al *confirmed the role of PG490 in apoptosis and went further to highlight that mitochondria, and not the death receptor, predominate in PG490 activity in mouse embryonic fibroblasts, due to caspase-9 activation [48]. This contradicted the findings of Frese *et al *where caspase-8 was found to be the key pro-enzyme in triggering apoptosis highlighting that mechanistic variability indeed exists with TCM as would be expected, thus caution must be taken when making any broad claims relating to their precise mechanism of action [42]. Synergistic induction of apoptosis has also been observed when chemotherapeutic agents are employed together with PG490, further corroborating the case for TCM use with conventional anticancer agents [48].

All cancer cells possess an elevated apoptotic threshold and although the therapeutic interventions of chemotherapy and γ-irradiation are crucial they are commonly plagued with resistance, resulting in a cycle of remission and relapse. The ever-rising incidence of resistance to chemotherapy suggests an increase in this apoptotic threshold and so the challenge is whether it can be reduced sufficiently to break the cycle at the point of remission. Advances made in recognizing and activating the key molecules involved in apoptotic pathways are certainly very encouraging from the perspective of eradicating a tumour although resistance to these interventions are also emerging [49]. The growing acceptance of TCM as a real adjunct therapy makes it an invaluable tool in the fight against many cancers and it holds much promise especially in cases where resistance to therapy is prevalent.

### Molecular basis of TCM – Angiogenesis

**Figure 3:**
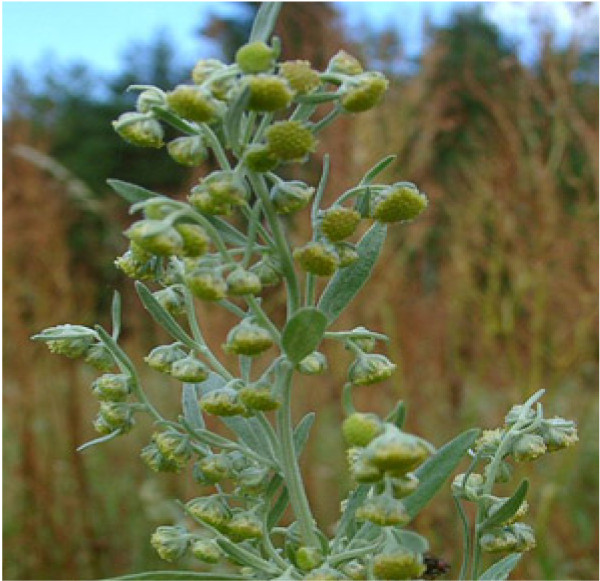
**Plant of *Artemisia absinthium *('Chinese wormwood')**.

**Figure 4:**
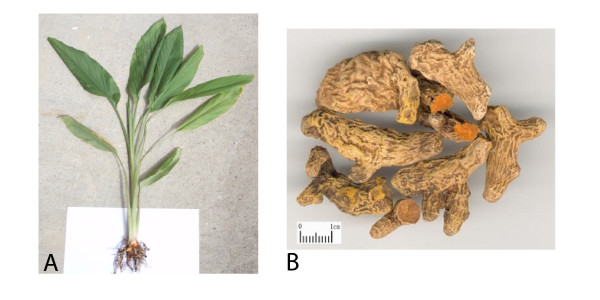
**Plant (A) and root (B) of *Curcuma longa *('curcumin') where root scale is shown as 1 cm**.

Angiogenesis – the creation of a healthy vascularised network by a tumour is a key underlying process in the induction and establishment of cancer [50]. It, like apoptosis, involves multi-step biochemical interactions that require activation of cell-signalling pathways, supply of nutrients and a host immune response. A range of TCM such as the Chinese wormwood (*Artemisia absinthium *– Figure 3), turmeric (*Curcuma longa *– Figure 4) and *Scutellaria Baicalensis *are commonly employed by traditional practitioners and studies demonstrate that their actions are at least in-part achieved by blocking the critical process of tumour vascularisation [51]. In order for cancer cells to grow and develop a healthy network of blood vessels high sources of nutrients and oxygen are vital. The rapidly dividing cells are subject to a hypoxic environment, so failure to set-up this fundamental framework results in stunted growth of the tumour (≤ 1–2 mm) and development of necrosis at its core [52,53]. Starving an established tumour of its blood supply involves an intervention in the complex angiogenic cascade, of which vascular endothelial growth factor (VEGF) is the most reported biomarker [54]. VEGF production is considered essential for angiogenesis and cancer metastasis, with high titres being indicative of a poor prognosis [55]. A wide array of oncogenes (e.g. *ras*, *HER-2*, *p53 *and *C-jun*) and growth factors (EGF, TGF, IGF and PDGF) have been identified as up-regulating VEGF-mRNA and so TCM that inhibits their expression and production, respectively, are also considered invaluable tools in cancer therapy.

Artemisinin, an active constituent of Chinese wormwood (*Artemisia absinthium *– Figure 3) is a potent antimalarial, however more recently it has been shown to possess anti-angiogenic properties, acting by lowering both VEGF and its receptor (VEGF-R2(in embryo), KDR(in humans) and flk-1(in mice)) in tumour and endothelial cells in a dose-dependent manner [56-59].

Phytochemicals have long been used as 'lead compounds' to generate drugs with better pharmacokinetic profiles and reduced toxicity *in vivo*. Artesunate (ART) and dihydro-ART are semi-synthetic derivatives of artemisinin with demonstrable activity against a wide range of cancer cell lines including KML-562 (chronic myeloid leukaemia), HeLa (cervical cancer) and HO-8910 (ovarian cancer) [60-62]. Human umbilical vein endothelial cells (HUVECs) are commonly employed alongside cancer cell assays to assess the extent to which angiogenesis is induced by way of new micro-vessel tube formation. Studies using this model show that dihydro-ART has significant anti-angiogenic activity compared to ART and prevents new-microvessel formation by 70–90% *in vitro *[61]. Along with the low toxicity profile associated with these agents their future role to complement treatment regimes is encouraging and warrants further investigation.

Curcumin (*Curcuma longa *– Figure 4), the principle curcuminoid in turmeric and widely used culinary spice is cytotoxic to cancer cells on a number of levels with a proven synergy when used in combination with chemotherapy/radiotherapy [63-65]. Its angioinhibitory action has been substantiated in a number of cancer cell lines including that of the breast where it was found to inhibit two major angiogenic factors, VEGF and b-FGF (basic-fibroblast growth factor) [66,67]. Besides this it also impedes tumour cell invasion, a property found to diminish circulating but not established metastases. It does this *via *downregulation of matrix metalloproteinases (MMP), most notably MMP-2 & MMP-9 – responsible for the invasive growth property of tumours [68-70]. In Ehrlich ascites tumour (EAT) cells a time-dependent inhibition of VEGF and key growth factor angiopoietin was observed, combined with an anti-proliferative effect on HUVECs, this being attributed directly to VEGF and NF-κB inhibition [71,72]. Other reported actions of curcumin include inhibition of epidermal growth factor receptor (EGFR) and intracellular signalling tyrosine kinases, the latter of which are known to promote angiogenesis through gene activation of cyclooxygenase-2, IL-2 and MMPs [73-75]. Derivatives of curcumin have also being investigated with preliminary findings pointing towards an increase in antitumor activity, although further corroborative studies are necessary to confirm these findings [76-78].

TCM often possess quite distinct and specialised modes of action, and consequently tumours normally resistant to conventional chemotherapy are reported to be more susceptible to TCM therapy [79]. They have demonstrable and often direct inhibitory effects on tumour cell growth and proliferation, affecting different stages of the cell growth cycle and mitotic phase [80]. Herbal compounds such as paclitaxel (Taxol^®^) and its derivatives suppress microtubule depolymerization, thus terminating cell mitosis [81]. As a result these, and compounds including harringtonine (*Cephalotaxus hainanensis*) and camptothecin (*Camptotheca acuminata*) with similar mechanisms of action are already commonly used in the clinic as anticancer agents for a variety of cancers [82]. The mechanism of action of camptothecin (*Camptotheca acuminata*) being to inhibit DNA topoisomerase I, consequently affecting DNA replication; paclitaxel is a mitotic spindle inhibitor (spindle poison), which can also bind with tubulin and prevent the normal physiological process of microtubule depolymerization.

**Figure 5:**
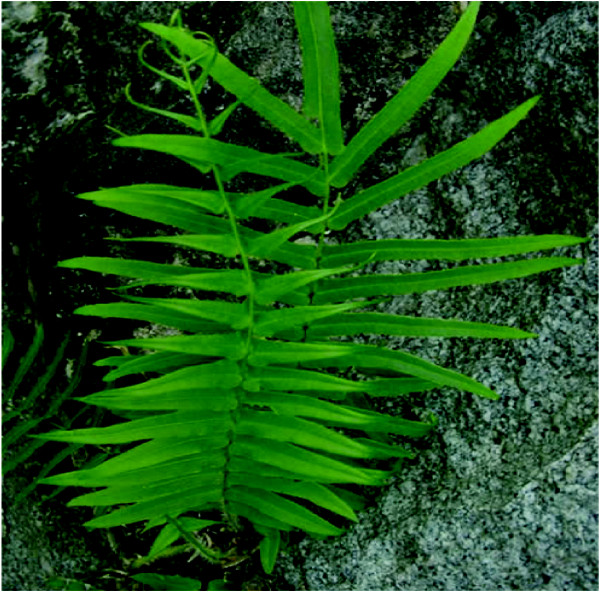
**Plant of *Pteris semipinnata L. *('half-flag')**.

Anticancer properties of the TCM elemene (*Rhizoma Zedoariae*) and oridonin (*Rabdosia rubescens*) lend themselves to being co-administered with conventional chemotherapeutic agents (e.g. doxorubicin and 5-fluorouracil (5-FU)) to impart a synergistic anti-tumour effect. Combining elemene (*Rhizoma Zedoariae*) with the pyrimidine base analogue 5-FU, resulted in significantly higher tumour growth inhibitive effects [83]. The anti-tumour activity of another TCM, 'half-flag' (*Pteris semipinnata L. *– Figure 5) was also significant, being shown to inhibit DNA production in HL-60 cells by 41% when combined with 5-FU, compared to only 10% in cells treated with the TCM alone. Half-flag also improved the anticancer efficiency of several other chemotherapeutic drugs when used concomitantly [84].

Similarly, derivatives of the herbal compound berbamine (*Berberis amurensis*), namely EBB (*O*-(4-ethoxyl-butyl)-berbamine) when combined with cyclophosphamide and mitomycin-C respectively enhanced the antitumor capacity, while also significantly improving patients' quality of life [85]. Zhang and co-workers tested 20 natural flavonoid compounds in breast cancer cell lines discovering they assist in the intracellular accumulation of anthracycline drugs while also reversing anthracene resistance [86,87]. Kim SW *et al*., reported that ginsenoside-Rg_3 _(*Panax ginsenoside Rg*_3_) promotes Rhodamine-123 accumulation in vincristine-resistant KBV20C human fibroblast cancer cells, reversing vincristine resistance acquired by a variety of cells [88].

The discovery that single components within TCM have the potential to overcome multidrug resistance developed by tumour cells opens the door to new avenues of multi-drug/TCM therapy. These findings justify and moreover pave the way for them to be used alongside conventional drugs, where significant resistance to therapy has already developed.

Although only one clinical trial using TCM was reported up until 2001 it was poorly controlled and any conclusions drawn were deemed unreliable, it is noteworthy however that given the very recent attention being received by TCM a re-analysis study has since been conducted [89,90]. This raises the call for more well-defined, robust and regulated clinical trials on TCM to ensure reliable data is generated which would enable regulatory authorities and clinicians alike to make well-informed decisions when considering their incorporation into western formularies.

## Conclusion

In treating diseases/illnesses of a systemic nature the typical route of TCM administration is oral. This is deemed a far from optimal approach in cancer patients given patient-to-patient variability when formulating TCM, the unpredictable absorption profile of the various bioactives across the gastrointestinal tract together with compliance issues. Furthermore, the challenge of ensuring batch-to-batch reproducibility of any given formulation remains, although the adoption of cutting-edge genetic/chemical fingerprinting in conjunction with micro-array-based cell line testing offers unique solutions to this. With modern techniques of isolation, characterisation and functionalisation of compounds along with *in vitro*/*in vivo *testing now common place within research facilities where drug discovery and delivery is a focus, the drive to engineer well-defined, targeted drug delivery systems offers a new dawn for this very traditional practice of medicine [91-93]. By identifying potent bioactives derived from TCM as discussed above, and tailoring formulations that encapsulate/incorporate them into cutting-edge drug delivery systems for parenteral administration one can envision overcoming the shortfalls that have prevented TCM being accepted by the West as a real adjunct/alternative to conventional cancer therapies. With a library of over 250,000 individual therapeutic compounds at our disposal, many of which have yet to be successfully isolated and tested for both safety and efficacy, there are certainly challenges that lay ahead – the scope and scale of which could well revolutionise drug discovery and delivery in the fight against cancer, for many decades to come.

## Competing interests

The authors declare that they have no competing interests.

## Authors' contributions

HSP drafted and wrote the manuscript. MQW revised the manuscript critically for important and intellectual content. GL provided translation of articles written in Chinese and revised the manuscript for intellectual content. All authors read and approved the final manuscript.
